# Sustainable tourism development for traditional Chinese drama's intangible cultural heritage

**DOI:** 10.1016/j.heliyon.2024.e25483

**Published:** 2024-01-30

**Authors:** Xi Zhao, Ehsan Elahi, Fushuai Wang, Hu Xing, Zainab Khalid

**Affiliations:** a School of Communication Sciences and Arts, Chengdu University of Technology, Chengdu, 610051, China; b School of Economics, Shandong University of Technology, Zibo, 255022, China; c Moscow School of Economics, Lomonosov Moscow State University, Moscow, 119992, Russia; d School of Humanities, Geely University of China, Chengdu, 641423, China; e School of Economics and Management, Southeast University, Nanjing, China

**Keywords:** Cultural heritage, Tourism, Sustainable development, Analytic hierarchy process, China

## Abstract

This study establishes an expert-driven evaluation system to assess the sustainable tourism development of drama-related intangible cultural heritage in China. Utilizing the Saaty 1–9 scale and hierarchical analysis method, 52 experts determined indicator weights and current development levels. Four dimensions are evaluated: humanistic value, project quality, tourism development, and sustainability. Results reveal humanistic value as most vital at 41.70 % weight. Secondary factors are project quality (29.89 %), tourism development (20.87 %), and sustainability (7.54 %). Aesthetic value, dissemination degree, and location conditions proved to the crucial tourism indicators. The ideological value of drama is paramount, alongside visibility and climate factors. The evaluation demonstrated strong preservation of humanistic value but deficiencies in tourism development, especially regarding infrastructure. Key recommendations include balancing preservation, dissemination, and innovation; emphasizing ideological value, visibility, and climate suitability; maintaining humanistic diversity; and improving site infrastructure. Further testing of evaluation indicators across periods is warranted alongside examining green revitalization potential. This assessment, guided by experts, offers a thorough framework for the sustainable development and preservation of the precious intangible heritage embodied in Chinese drama.

## 1 Introduction

Intangible cultural heritage, deeply rooted in the practices of people, stands as a vital testament to the history and identity of a race, acting as a nation's emblematic image and brand. Within this broad spectrum, traditional drama occupies a prominent place, representing 12 % of China's listed intangible cultural heritage. This includes culturally rich art forms like Kunqu Opera, Cantonese Opera, Shadow Opera, and Peking Opera [1]. Traditional drama's intangible heritage, with its ease of appreciation and popularity, makes it an attractive option for tourism development [2].

In the vast cultural landscape of China, Sichuan, and Chongqing are regions of significant importance regarding intangible cultural heritage. These areas are unique as they encompass all ten categories of intangible cultural heritage. Notably, traditional drama is the most abundant form of intangible cultural heritage in these regions [3]. The rich tapestry of ethnic diversity in these areas, including ethnic groups like the Yi, Miao, Hui, and Tibetan, adds to the unique cultural beliefs and practices. The existence of these diverse communities enhances the region's distinct cultural identity, as evidenced by the clustered distribution pattern of this heritage across multiple cities and prefectures such as Liangshan, Aba, and Ganzi, as observed by McLaren [4].

Despite its richness, the intangible cultural heritage of drama in Sichuan and Chongqing faces several challenges. The traditional modes of oral and family-based transmission are becoming less effective due to modern developmental influences, leading to a decline in the attention and preservation of this heritage [5]. Additionally, the influence of drama-related intangible cultural heritage is limited, with only a few projects having a national impact, leaving others less recognized [6]. Another concern lies in the monotony of disseminating drama heritage, which falls short of meeting the audience's yearning for diverse and enriching experiences [7].

The current study along with various sociological studies, suggests that the development of tourism centered on intangible cultural heritage can enhance its dissemination and visibility. This not only improves the influence of national culture but also enriches tourists' experiences through diverse forms of expression [[8], [9], [10], [11]]. However, it is crucial to establish a sustainable development model for the tourism promotion of drama-related intangible cultural heritage. A balanced approach is needed to prevent dilemmas such as underdevelopment or excessive commercial exploitation, which could undermine the intrinsic human spirit and authenticity of these cultural projects [12].

To address these challenges, the study proposes the establishment of a comprehensive evaluation and guidance system, specifically tailored for the tourism development of intangible cultural heritage in the domain of drama in southwest China. The methodology involved a hierarchical sampling survey and in-depth interviews with experts from the fields of intangible cultural heritage of drama and tourism. The comprehensive integration of these discussions and analyses has intricately shaped both the structure and content of our study. The study is systematically divided into five parts. The first section delves into the background and the significance of the study. The second discusses the literature review and the novel aspects of this study. The third outlines the data collection, evaluation indicators, and research process. The fourth section involves the computation and analysis of indicator weights and evaluations. Similarly, the fifth and final section presents the key conclusions and future directions for the development of intangible cultural heritage tourism with a focus on drama.

## 2 Literature review and contributions of the study

Previous studies on intangible cultural heritage tourism of drama have focused on two key aspects. The first major aspect involves investigating various approaches to effectively leverage and promote the development of drama-related heritage resources for tourism purposes [13]. The second vital aspect centers around examining and formulating strategies to ensure the safeguarding and preservation of existing tourism resources associated with drama intangible cultural heritage.

When delving into these two dimensions of drama heritage tourism research, notable differences arise in the perspectives and methodologies employed by Chinese scholars in contrast to researchers from various countries worldwide. Several factors may account for these variations, including divergent cultural outlooks, regional developmental priorities, and differing frameworks regarding the preservation and promotion of intangible cultural heritage [14].

Firstly, Chinese studies emphasized on practical investigative aspects concerning drama heritage tourism resources. There is a focus on elucidating the tangible spatial distribution characteristics and influencing elements shaping these resources. For instance, aspects like national heritage attributes [15], spiritual values [16], and foundational resource development [17] have been examined. Moreover, research spans various levels ranging from specific regional analyses to nationwide statistical mappings representing the overall national situation [18]. There is also a macro-level interest in illuminating the role of dramatic heritage in broader economic operations and development [[19], [20], [21], [22], [23]].

Secondly, in terms of research methods, Chinese academics prefer to concentrate their studies using micro-level units of analysis as the targets of examination. The emphasis is on teasing apart the latent or underutilized tourism development potential for specific examples of intangible cultural heritage related to the drama [24]. International efforts differ by taking a broader approach focused on first identifying overarching practical pathways. Accordingly, various conceptual models have been constructed seeking to encapsulate frameworks applicable across cases. Examples include establishing practical theories like the RPM model, alongside technical tools such as Geographic Information System models that integrate spatial mapping [19,25,26].

Regarding perspectives on safeguarding existing tourism resources tied to drama intangible cultural heritage, there appears to be a consistent consensus between Chinese and international scholars. A two-pronged stance is widely promoted – firstly, according to special care and favorable protections towards examples of drama heritage. Secondly, ensure any initiatives to develop associated tourism resources operate within imposed limitations and regulations to prevent the destruction of the foundational heritage [27,28]. Thus, the premise lies in cautiously balancing the preservation of core authenticity and essence alongside allowing for sustainable scales of tourism-based development [29,30]. Additionally, academics in China and abroad have put forth several corresponding recommendations regarding concrete measures on how to safeguard dramatic intangible cultural heritage. Areas of alignment include the necessity of government guidance, mobilizing social forces, enacting comprehensive protection policies, and enhancing the inherent “self-blooding” function embedded within promising drama heritage examples [[31], [32], [33]].

While existing literature emphasizes the desire to promote tourism development tied to drama intangible cultural heritage within appropriate conservation-minded frameworks, many proposals remained at a superficial conceptual level. There is a lack of properly delineated implementation plans, programs, or adequately rigorous and sustainable evaluation systems to assist realization. This study therefore aims to address this underexplored research area through a pioneering quantitative investigation focused on revealing synergistic mechanisms between drama heritage preservation and strategic tourism cultivation. Expert surveys will guide the customized construction of a comprehensive indicators-based evaluation system to shape future practices. By transforming initially subjective assessments into hierarchical analytical models, the aim is to establish a more objective, robust, and reliable framework for decision-making to steer tourism development policies and enhance this invaluable cultural realm. The main contributions of this study to the field of hospitality and tourism management, particularly in the realm of sustainable tourism development for traditional Chinese drama's intangible cultural heritage are as follows:

1. Comprehensive Evaluation System: By utilizing the Saaty scale value method and hierarchical analysis, we have established a comprehensive and scientific evaluation system with objective quantitative scores for sustainable tourism development of traditional Chinese drama's intangible cultural heritage. This system provides a practical framework for stakeholders, ensuring a more objective and well-informed approach to tourism development.
2. Identification of Key Indicators: The study identifies and quantifies the importance of several key primary and secondary indicators for sustainable tourism development related to the intangible cultural heritage of drama. Notably, the humanistic value of drama, aesthetic value, dissemination degree, and location conditions are established as crucial elements.
3. Empirical Evidence: Based on data collected in the regions of Sichuan and Chongqing, we provide empirical evidence validating the importance of these indicators. The findings reveal the pivotal role of location in attracting tourists and suggest off-site development as a viable strategy.
4. Holistic Approach: The study advocates a comprehensive, sustainable approach to tourism development focusing on humanistic and aesthetic values, enhancing visibility, and improving infrastructure.
5. Solution to Challenges: The study proposes sustainable tourism development as a solution to the challenges currently faced in the transmission, dissemination, and innovation of traditional Chinese drama's intangible cultural heritage.
6. Guide for Future Research and Implementation: The insights derived from this research can be instrumental for future studies in the field and guide policymakers, stakeholders, and practitioners in developing effective strategies for sustainable tourism linked with drama-related intangible cultural heritage.
7. Contributions to Literature: The study contributes to the existing literature by addressing a significant gap - it provides a detailed plan and evaluation system for the promotion of drama-related intangible cultural heritage tourism within the context of conservation efforts. The study moves beyond the superficial discussion of the concept in existing literature and presents a well-defined, practical framework.

## 3 Materials and methods

### 3.1 Study area and data collection

Sichuan Province and Chongqing City in China were selected as the study sites for this investigation into sustainable tourism development for drama-related intangible cultural heritage. These regions contain a prolific abundance and diversity of preserved examples of Chinese dramatic heritage. Data from the Ministry of Culture and Tourism shows Sichuan has 189 identified provincial-level intangible cultural heritage initiatives, while Chongqing hosts 62 such projects. The dramatic 3:1 ratio between the regions provided an ideal foundation to examine this tourism phenomenon. Moreover, the density of historical theater-focused heritage examples across Sichuan and Chongqing offers unparalleled access to understanding the dynamics between cultural conservation through dramatic arts and strategic tourism cultivation.

By the approximated 3:1 ratio of intangible cultural heritage projects between Sichuan and Chongqing, 52 experts spanning these regions were selected for in-depth surveys and interviews. The sampling methodology ensured that collected perspectives were informed by interdisciplinary expertise, encompassing domains like economics, tourism management, ethnography, and drama-focused cultural heritage administration. This afforded multidimensional insights, reflecting the complex challenges of balancing the preservation of an ancient art form with commercial tourism development. The notion of sustainability guiding expert inquiries similarly absorbed multiple facets, from maintaining authentic transmission of live theater practices to wisely safeguarding the surrounding natural environments.

A meticulous, two-step data-gathering process was implemented based on these expert consultations. The first phase focused on determining Saaty values that represent the weights experts assign to each performance indicator within the overall tourism assessment system (Table 1). Elucidating relative dimensional importance offers crucial guidance in planning initiatives and directing resources. The second analytical step centered on evaluating the status of tourism development initiatives for regional drama-based intangible cultural heritage post-establishment of the weighted evaluation architecture. Expert ratings were converted into quantitative metrics to enable nuanced comparisons of progress toward sustainability across projects. Throughout, judgment matrices based on Saaty input ratings underwent stringent consistency testing with only credible results retained for investigation.

**Table 1:** Statistics on the number of tested and available judgment matrices.

Judgment Matrix	N	Judgment Matrix	N	Judgment Matrix	N
$\left[\begin{array}{ccc} 1 & \cdots & a_{1,4}\\ \vdots & \ddots & \vdots \\ \frac{1}{a_{1,4}} & \cdots & 1 \end{array}\right]$	47	$\left[\begin{array}{ccc} 1 & \cdots & a_{11,14}\\ \vdots & \ddots & \vdots \\ \frac{1}{a_{11,14}} & \cdots & 1 \end{array}\right]$	47	$\left[\begin{array}{cc} 1 & a_{111,112}\\ \frac{1}{a_{111,112}} & 1 \end{array}\right]$	50
				$\left[\begin{array}{ccc} 1 & \cdots & a_{121,124}\\ \vdots & \ddots & \vdots \\ \frac{1}{a_{121,124}} & \cdots & 1 \end{array}\right]$	48
				$\left[\begin{array}{ccc} 1 & a_{131,132} & a_{131,133}\\ \frac{1}{a_{131,132}} & 1 & a_{132,133}\\ \frac{1}{a_{131,133}} & \frac{1}{a_{132,133}} & 1 \end{array}\right]$	47
				$\left[\begin{array}{cc} 1 & a_{141,142}\\ \frac{1}{a_{141,142}} & 1 \end{array}\right]$	50
		$\left[\begin{array}{ccc} 1 & \cdots & a_{21,24}\\ \vdots & \ddots & \vdots \\ \frac{1}{a_{21,24}} & \cdots & 1 \end{array}\right]$	47	$\left[\begin{array}{cc} 1 & a_{211,212}\\ \frac{1}{a_{211,212}} & 1 \end{array}\right]$	50
				$\left[\begin{array}{cc} 1 & a_{221,222}\\ \frac{1}{a_{221,222}} & 1 \end{array}\right]$	50
				$\left[\begin{array}{cc} 1 & a_{231,232}\\ \frac{1}{a_{231,232}} & 1 \end{array}\right]$	50
				$\left[\begin{array}{ccc} 1 & a_{241,242} & a_{241,243}\\ \frac{1}{a_{241,242}} & 1 & a_{242,243}\\ \frac{1}{a_{241,243}} & \frac{1}{a_{242,243}} & 1 \end{array}\right]$	48
		$\left[\begin{array}{ccc} 1 & a_{31,32} & a_{31,33}\\ \frac{1}{a_{31,32}} & 1 & a_{32,33}\\ \frac{1}{a_{31,33}} & \frac{1}{a_{32,33}} & 1 \end{array}\right]$	48	$\left[\begin{array}{ccc} 1 & a_{311,312} & a_{311,313}\\ \frac{1}{a_{311,312}} & 1 & a_{312,313}\\ \frac{1}{a_{311,313}} & \frac{1}{a_{312,313}} & 1 \end{array}\right]$	47
				$\left[\begin{array}{ccc} 1 & \cdots & a_{321,324}\\ \vdots & \ddots & \vdots \\ \frac{1}{a_{321,324}} & \cdots & 1 \end{array}\right]$	47
				$\left[\begin{array}{ccc} 1 & \cdots & a_{331,335}\\ \vdots & \ddots & \vdots \\ \frac{1}{a_{331,335}} & \cdots & 1 \end{array}\right]$	47
		$\left[\begin{array}{ccc} 1 & \cdots & a_{41,44}\\ \vdots & \ddots & \vdots \\ \frac{1}{a_{41,44}} & \cdots & 1 \end{array}\right]$	49	$\left[\begin{array}{ccc} 1 & a_{411,412} & a_{411,413}\\ \frac{1}{a_{411,412}} & 1 & a_{412,413}\\ \frac{1}{a_{411,413}} & \frac{1}{a_{412,413}} & 1 \end{array}\right]$	47
				$\left[\begin{array}{ccc} 1 & a_{421,422} & a_{421,423}\\ \frac{1}{a_{421,422}} & 1 & a_{422,423}\\ \frac{1}{a_{421,423}} & \frac{1}{a_{422,423}} & 1 \end{array}\right]$	49
				$\left[\begin{array}{ccc} 1 & a_{431,432} & a_{431,433}\\ \frac{1}{a_{431,432}} & 1 & a_{432,433}\\ \frac{1}{a_{431,433}} & \frac{1}{a_{432,433}} & 1 \end{array}\right]$	47
				$\left[\begin{array}{ccc} 1 & \cdots & a_{441,444}\\ \vdots & \ddots & \vdots \\ \frac{1}{a_{441,444}} & \cdots & 1 \end{array}\right]$	48

Overall, this dual dataset encompassing dimension weights and project development levels afforded an invaluable opportunity. Experts illuminated pathways forward through a complex terrain at the intersection of safeguarding ancient, yet living, dramatic arts practices and harnessing tourism for sustainable futures. Balancing economic livelihoods with maintaining the essence of cultural heritage stands as a profound challenge worldwide. By rigorously quantifying expert guidance, this investigation offers a replicable decision-making toolkit for policymakers and communities seeking prosperity while retaining the wisdom of the past.

### 3.2 Quantification of survey

Before conducting hierarchical analysis calculations, the experts' questionnaire needed quantification. Experts are to compare the importance of indicators at the same level. We used the Saaty 1–9 scale to convert their assessments into numbers [34]. Initially, we calculate the average difference in the indicator system's scores. Then, using consensus criteria for this mean difference, we assign numerical values to the experts' textual opinions on each indicator. Table 2 displays the match between these values, the mean differences (A_ij_), and the questionnaire options' significance.

**Table 2:** Saaty 1–9 scale values.

Values	Mean difference (A_ij_)	Meaning of the questionnaire options
1	A_ij_ = 0	Factor i is as important as factor j
3	0.25<A_ij_≤0.50	Factor i is slightly more important than factor j
5	0.75<A_ij_≤1.00	Factor i is significantly more important than factor j
7	1.25<A_ij_≤1.50	Factor i is strongly more important than factor j
9	1.75<A_ij_	Factor i is extremely more important than factor j
2, 4, 6, 8	Intermediate values of 1–3, 3–5, 5–7 and 7-9	

### 3.3 Weight calculation

The fundamental aspect of the hierarchical analysis method involves creating the judgment matrix (Salamzadeh et al., 2021). Designate the judgment matrix as *A*. The next steps include calculating the eigenvalues and eigenvectors of *A*. Let *λ* represent the maximum eigenvalue of *A*, and *X* denotes the corresponding eigenvector. The components of *X*, denoted as $X_{i}$*,* represent the weights assigned to the corresponding factors. We have normalized the column vectors of matrix A using equation (1).

(1) $$\bar{a_{ij}}=\frac{a_{ij}}{\sum_{k=1}^{n}a_{kj}}\;\left(i,j=1,2,3,\ldots ,n\right)$$

where $\bar{a_{ij}}$ is the judgment matrix that is normalized. In the next step, $\bar{a_{ij}}$ is summed by rows, as given in equation (2).

(2) $$\bar{x_{i}}=\sum_{j=1}^{n}\bar{a_{ij}}\;\left(i=1,l2,l3,\ldots ,l\;n\right)$$

where $\bar{x_{i}}$ denotes the numerical elements after summing by rows. The column vector obtained after summing by rows is normalized using equation (3).

(3) $$x_{i}=\frac{\bar{x_{i}}}{\sum_{i=1}^{n}\bar{x_{i}}}\;\left(i=l1,2,l\;3,\ldots ,ln\right)$$

where $x_{i}$ denotes the element obtained after the normalization process. The final desired Eigenvector *X* and the maximum Eigenvalue are obtained using equations (4), (5)).

(4) $$X={\left[x_{1},x_{2},x_{3},\cdots ,x_{n}\right]}^{T}$$

(5) $$\lambda =\sum_{i=1}^{n}\frac{{\left(AX\right)}_{i}}{nX_{i}}\;\left(i=1,2,3,\ldots ,n\right)$$

### 3.4 Consistency test

Subjective factors can affect experts' judgments, leading to potential inconsistencies. To mitigate this, consistency checks are crucial. We conducted a reliability test using three methods: the placebo test (SMC), Cronbach's α, and the CR metric. Following Abadie et al. [35] and Spearman [36], we used (SMC) and Cronbach's α to determine the reliability of the data. Moreover, CR statistic was determined using equations (6), (7)). The consistency indicator CI was calculated using equation (6).

(6) $$CI=\frac{\lambda -n}{n-1}$$

where n is the order of the matrix. The greater the value of *CI*, the poorer the consistency between *A* and the less coordination it exhibits. Further, we found the stochastic consistency ratio CR using equation (7).

(7) $$CRl=l\frac{\text{CI}}{RI}$$

where RI is the average random consistency index of the same order. Usually, the results are considered to have good consistency only when CR<0.1. The confidence intervals for the values of each indicator adopted in this study are given in Table 3.

**Table 3:** Permissible value intervals for the values of the statistics.

Test	St. Value
SMC	≥0.5
Cronbach's α	≥0.7
CR	＜0.1

### 3.5 Indictors framework

The study evaluates sustainability and long-term viability of “drama-related intangible cultural heritage and tourism.” Expert interviews were carefully conducted to formulate a comprehensive set of evaluation criteria. The profound cultural and societal significance of drama-related intangible cultural heritage contributes to historical understanding and the nurturing of cultural identity. Preserving the project's quality is crucial for safeguarding heritage, enhancing visitor enjoyment, and facilitating widespread dissemination. The tourism development of drama-related intangible cultural heritage is essential for both preservation and attracting tourists. Sustainability, in this context, encompasses the project's longevity, social and environmental responsibility, and a well-balanced business model aimed at upholding and revitalizing intangible cultural heritage [4,6,7,11,12,16,18].

The study emphasized four different aspects: humanistic value, project quality, tourism development, and sustainability. Each of these has its own set of observable variables. Under humanistic value, variables such as aesthetic, artistic, cultural, and entertainment values are considered, which include aspects like the ideological value of drama, the live experience of the show, text creation, stage presentation, the use of multimedia technology, subject matter features, leisure, and audience engagement. In the realm of project quality, the focus is divided into dissemination degree, creative team, safeguard facilities, and IP quality. This encompasses factors like visibility, reputation, the professionalism and management of the theatre team, the quality of both hardware and software facilities, and the untapped potential and depth of the intellectual property.

Tourism development is analyzed through variables like location conditions, supporting facilities, and development status. This includes considerations such as climate, economic development level, traffic and shopping convenience, dining and accommodation conditions, level of development, attendance rates, ticket pricing, audience demographics, and the effectiveness of the business model. Lastly, sustainability is assessed through variables relating to survival status, environmental responsibility, social responsibility, and business feedback. This covers a range of factors from preservation, inheritance, and innovation, to environmental protection, social ethics, public interest protection, sustainable profitability, and reinvestment in non-genetic heritage. Each indicator within this evaluation system is given a unique code, mentioned alongside its name. It provides a clear framework for understanding the interrelationships among these diverse yet interconnected factors (Table 4).

**Table 4:** Evaluation index system.

Tier 1 Indicators	Tier 2 Indicators	Tier 3 Indicators
Humanistic values (I_1_)	Aesthetic value (I_1, 1_)	The ideological value of drama (I_1, 1, 1_)
		The experience of the show (I_1, 1, 2_)
	Artistic value (I_1, 2_)	Text creation for drama (I_1, 2, 1_)
		Presentation of the stage (I_1, 2, 2_)
		Live nature of the show (I_1, 2, 3_)
		The use of multimedia technology (I_1, 2, 4_)
	Cultural values (I_1, 3_)	Features of the subject matter (I_1, 3, 1_)
		Historical culture (I_1, 3, 2_)
		Promotional value (I_1, 3, 3_)
	Entertainment value (I_1, 4_)	Leisure and entertainment (I_1, 4, 1_)
		Audience engagement (I_1, 4, 2_)
Project quality (I_2_)	Dissemination degree (I_2, 1_)	Visibility (I_2, 1, 1_)
		Reputation (I_2, 1, 2_)
	Creative team (I_2, 2_)	Professionalism of the team (I_2, 2, 1_)
		Management status of the theater (I_2, 2, 2_)
	Safeguard facilities (I_2, 3_)	Quality of hardware facilities (I_2, 3, 1_)
		Quality of software facilities (I_2, 3, 2_)
	IP quality (I_2, 4_)	A potential worth tapping (I_2, 4, 1_)
		A depth worth digging (I_2, 4, 2_)
		IP legacy degree (I_2, 4, 3_)
Tourism development (I_3_)	Location conditions (I_3, 1_)	Climatic conditions (I_3, 1, 1_)
		Location conditions (I_3, 1, 2_)
		Economic development level (I_3, 1, 3_)
	Supporting facilities (I_3, 2_)	Traffic convenience (I_3, 2, 1_)
		Shopping convenience (I_3, 2, 2_)
		Conditions for dining (I_3, 2, 3_)
		Conditions of the accommodation (I_3, 2, 4_)
	Development status (I_3, 3_)	Level of development (I_3, 3, 1_)
		Attendance rate (I_3, 3, 2_)
		Reasonableness of ticket prices (I_3, 3, 3_)
		Audience demographic attributes (I_3, 3, 4_)
		Virtuous business model (I_3, 3, 5_)
Sustainability (I_4_)	Survival status (I_4, 1_)	Preservation (I_4, 1, 1_)
		Inheritance (I_4, 1, 2_)
		Innovation (I_4, 1, 3_)
	Environmental responsibility (I_4, 2_)	Protection of the environment (I_4, 2, 1_)
		Compensation for the environment (I_4, 2, 2_)
		Recovery after damage (I_4, 2, 3_)
	Social responsibility (I_4, 3_)	Security (I_4, 3, 1_)
		Social Ethics (I_4, 3, 2_)
		Protection of public interest (I_4, 3, 3_)
	Business feedback (I_4, 4_)	Sustainable profitability (I_4, 4, 1_)
		Sustainable income for the heir (I_4, 4, 2_)
		Reinvesting in non-genetic heritage (I_4, 4, 3_)
		Reinvestment in the development of non-heritage (I_4, 4, 4_)

### 3.6 Distinctness test

To mitigate the potential negative impact of subjectivity on the design of evaluation system indicators, differentiation validity tests were conducted. The average extracted variance (AVE) and Kaiser-Meyer-Olkin (KMO) statistics were employed for the discriminant test of variables. AVE, measured across each group of matrices, exceeded the criterion of 0.5. Although not all KMO values crossed 0.7, they were greater than 0.6. According to the applicability scale of KMO, the variables in this study exhibit significant differentiation, indicating that the index system setting is reasonably appropriate.

## 4 Results and discussion

### 4.1 Determining the relative importance of sustainability indicators

To evaluate the multitude of potential factors influencing the sustainable tourism development of drama intangible cultural heritage, experts quantified the relative importance of various indicators through a two-phase weighting process (Table 5).

**Table 5:** Evaluation index system with integrated weights.

Tier 1 Indicators	Separate weights	Tier 2 Indicators	Separate weights	Combined weights	Tier 3 Indicators	Separate weights	Combined weights
I_1_	0.4170	I_1, 1_	0.5138	0.2142546	I_1, 1, 1_	0.8099	0.173525
					I_1, 1, 2_	0.1901	0.040730
		I_1, 2_	0.2612	0.1089204	I_1, 2, 1_	0.5122	0.055789
					I_1, 2, 2_	0.2773	0.030204
					I_1, 2, 3_	0.1289	0.014040
					I_1, 2, 4_	0.0816	0.008888
		I_1, 3_	0.1366	0.0569622	I_1, 3, 1_	0.6182	0.035214
					I_1, 3, 2_	0.2663	0.015169
					I_1, 3, 3_	0.1155	0.006579
		I_1, 4_	0.0885	0.0369045	I_1, 4, 1_	0.8442	0.031155
					I_1, 4, 2_	0.1558	0.005750
I_2_	0.2989	I_2, 1_	0.4961	0.1482843	I_2, 1, 1_	0.8204	0.121652
					I_2, 1, 2_	0.1796	0.026632
		I_2, 2_	0.2796	0.0835724	I_2, 2, 1_	0.7790	0.065103
					I_2, 2, 2_	0.2210	0.018470
		I_2, 3_	0.1604	0.0479436	I_2, 3, 1_	0.7883	0.037794
					I_2, 3, 2_	0.2117	0.010150
		I_2, 4_	0.0639	0.0190997	I_2, 4, 1_	0.6256	0.011949
					I_2, 4, 2_	0.2656	0.005073
					I_2, 4, 3_	0.1087	0.002076
I_3_	0.2087	I_3, 1_	0.6253	0.1305001	I_3, 1, 1_	0.6398	0.083494
					I_3, 1, 2_	0.2729	0.035613
					I_3, 1, 3_	0.0873	0.011393
		I_3, 2_	0.2689	0.0561194	I_3, 2, 1_	0.4733	0.026561
					I_3, 2, 2_	0.2880	0.016162
					I_3, 2, 3_	0.1666	0.009349
					I_3, 2, 4_	0.0721	0.004046
		I_3, 3_	0.1058	0.0220804	I_3, 3, 1_	0.4877	0.010769
					I_3, 3, 2_	0.2126	0.004694
					I_3, 3, 3_	0.1716	0.003789
					I_3, 3, 4_	0.0877	0.001936
					I_3, 3, 5_	0.0404	0.000892
I_4_	0.0754	I_4, 1_	0.5400	0.0407160	I_4, 1, 1_	0.6597	0.026860
					I_4, 1, 2_	0.2494	0.010155
					I_4, 1, 3_	0.0909	0.003701
		I_4, 2_	0.2492	0.0187897	I_4, 2, 1_	0.5655	0.010626
					I_4, 2, 2_	0.2866	0.005385
					I_4, 2, 3_	0.1479	0.002779
		I_4, 3_	0.1295	0.0097643	I_4, 3, 1_	0.6499	0.006346
					I_4, 3, 2_	0.2482	0.002423
					I_4, 3, 3_	0.1019	0.000995
		I_4, 4_	0.0813	0.0061300	I_4, 4, 1_	0.5496	0.003369
					I_4, 4, 2_	0.2430	0.001490
					I_4, 4, 3_	0.1409	0.000864
					I_4, 4, 4_	0.0664	0.000407

Firstly, at the indicator group level in Tier 1, humanistic value emerged as the most crucial high-level element according to experts, carrying 41.7 % weight. This suggests that the cultural essence and underlying values that distinguish this specific art form must be preserved for drama-focused tourism to be sustainable. Project quality followed closely behind at 29.89 %, highlighting the need for productions and performances of adequate creative caliber and management rigor to attract ongoing tourism. Tourism development itself was weighted at 20.87 %, speaking to the commercial elements required for financial sustainability. Though rated least important, sustainability at 7.54 % weight still carried meaning, encompassing ecological impacts.

Secondly, within the most vital humanistic value grouping in Tier 2, aesthetic appeal manifested as the foremost specific indicator at 51.38 % weight. This indicates that leveraging the innate aesthetic artistry and beauty unique to Chinese theater arts serves as the prime channel for visitor attraction and tourism competitiveness. Comparatively, entertainment value lagged significantly lower at 8.85 %. However, after computing its combined weight incorporation relationships with higher tiers, entertainment climbed to 3.69 % influence. This suggests that while secondary, the ability for interactive enjoyment remains relevant.

Outcomes were similar for other indicator dimensions. In project quality, separate weights showed the creative team's professional expertise and administrative leadership dominated at 49.61 %, while IP potential and depth contribution lagged at 6.39 %. Nonetheless, combined weights revealed a more balanced influence between these secondary markers of production quality and marketability. Comparatively assessing weights for tourism development and sustainability illuminated crucial spatial, commercial, environmental, and managerial factors.

By accounting for experts’ stratified weighting evaluations, precise guidance emerges on key leverage points spanning policy programs, infrastructure investments, marketing activities, and development practices. The insights center on maintaining the humanistic essence and production vitality of regional dramatic arts by emphasizing aesthetics and location alongside strategic integration of commercial entertainment platforms.

### 4.2 Key elements for sustainable drama heritage tourism

**Fig. 1:**
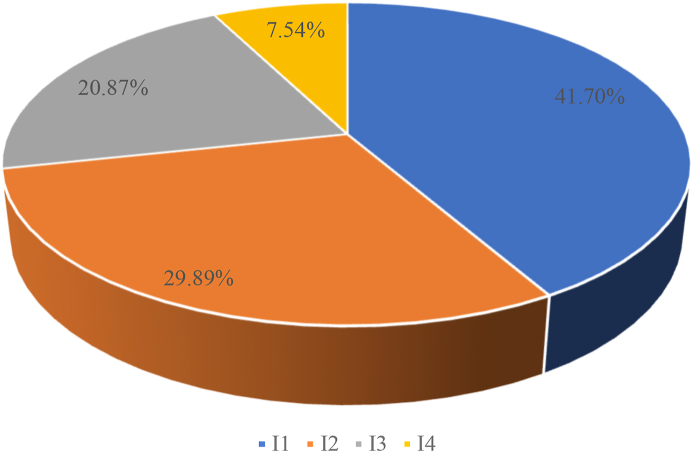
The weighting of first-level indicators.

The weighted analysis indicates that humanistic value ranked most critical for sustainable drama heritage tourism at 41.70 % importance (Fig. 1). This supports Smith and Campbell's argument [37] that humanistic value signifies a theater's appreciation for the foundational aspects of humanity. Additionally, it resonates with the findings of Brown [38]. Preserving and enriching drama's cultural depth remains vital. Project quality holds the second-highest weight at 29.89 %. Similarly, Kim et al. [11] emphasized theater production value for visitor satisfaction and sustainability. Ensuring creative robustness, management integrity, and the presence of social media remained key to destination competitiveness [39].

Tourism development follows with a 20.87 % weight. As Levchenko et al. [40] articulated, this encompasses strategic improvements to leverage heritage assets for tourism. Though secondary in weighting, tourism sustainability, and environmental protection considerations cannot be dismissed, as argued in many studies [39,[41], [42], [43]]. More symbiotic integration between heritage preservation efforts and commercial tourism vectors, as promoted by Kim [11], may require consideration to avoid over-commercialization diluting core heritage value.

While tourism integration proves essential for financial viability, safeguarding the humanistic essence and internal vitality inherent in the art's practice should take utmost priority for enabling sustainable futures. Ongoing research efforts must focus on identifying optimal balances where this ancient performative legacy can simultaneously retain its integrity yet reach wider communities through thoughtful dissemination channels.

### 4.3 Expanding drama-related intangible cultural heritage tourism: key indicators and strategic development

**Fig. 2:**
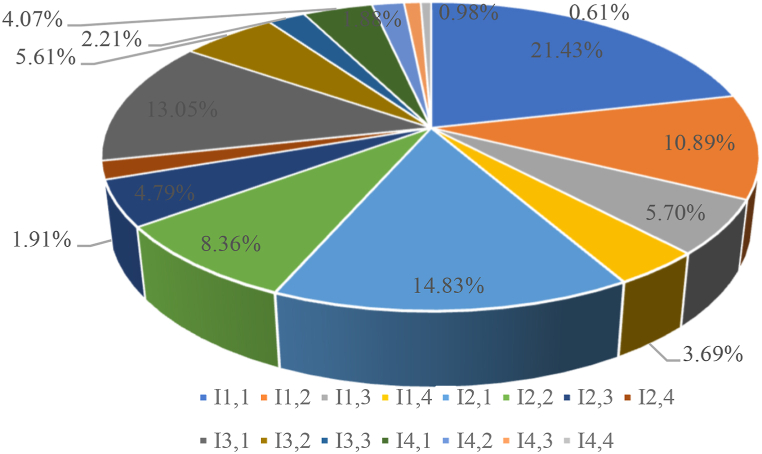
Weights of second-level indicators.

Fig. 2 identifies three key secondary indicators critical to drama-related intangible cultural heritage tourism: aesthetic value (21.43 %), dissemination degree (14.83 %), and location conditions (13.05 %). Experts, such as Cochrane [44] and Geraghty [45], stress the importance of showcasing the distinct aesthetic features of drama projects in tourism development. The importance of expanding dissemination for the sustainability of intangible cultural heritage tourism is backed by research focusing on Greek drama and cultural activism [46,47]. The role of location in attracting tourists is also highlighted, with its significant influence on the number of local visitors as shown by Erkin and Shakhrizoda [48].

Given the reliance of drama-related intangible cultural heritage tourism on theaters for highlighting performances, there is a suggestion to pivot towards off-site development. This strategy involves extending cultural experiences and performances beyond conventional theater environments, thus reaching a more diverse audience, and enhancing engagement with this unique form of intangible heritage. Prioritizing off-site development not only improves accessibility but also draws a broader spectrum of visitors. This approach is instrumental in both preserving and promoting drama-related intangible cultural heritage.

### 4.4 Key influencers in drama heritage tourism

**Fig. 3:**
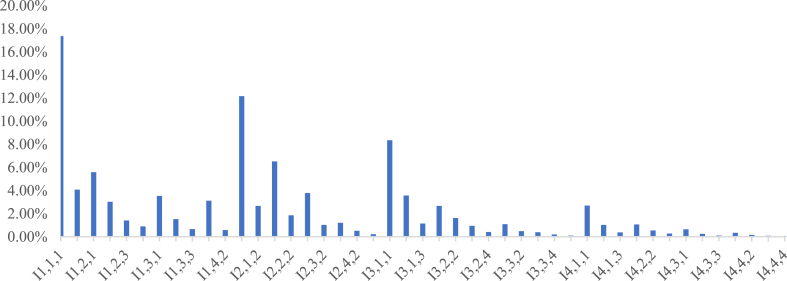
Weights of third-level indicators.

Fig. 3 highlights the prominent roles of ideological value in drama, visibility, and climatic conditions at theaters in drama-related intangible cultural heritage tourism, carrying weights of 17.35 %, 12.17 %, and 8.35 % respectively. The ideological value is the foremost element that sets drama-related intangible cultural heritage apart, enhancing its appeal in tourism development. This inherent cultural aspect underscores the heritage's distinctiveness, attracting tourists seeking genuine and profound experiences. Visibility, critical for widespread dissemination and effective communication, and climatic conditions, which shape regional traits and impact tourism development, are also significant factors requiring consideration when assessing drama heritage sustainability [[49], [50], [51]].

### 4.5 Evaluation of sustainable tourism development for drama-related intangible cultural heritage

In this study, fifty-two experts in sustainable tourism and intangible cultural heritage completed a survey rating three-level indicators gauging current sustainable tourism development status for Southwest Chinese dramatic heritage sites. They assigned scores from 1 to 10 to each indicator. Table 6 shows the descriptive statistics and weighted comprehensive scores after excluding the 1 % and 99 % outliers from the original data. The evaluation revealed that among the four dimensions assessed - humanistic value, project quality, tourism development, and sustainability - experts viewed the humanistic value dimension most favorably regarding current sustainable tourism development for dramatic intangible cultural heritage sites.

**Table 6:** Current status score.

Tier 1 Indicators	Score	Tier 2 Indicators	Score	Tier 3 Indicators	Score	Standard Deviation	Maximum	Minimum
I_1_	8.26	I_1, 1_	8.36	I_1, 1, 1_	8.40	1.56	10.00	6.00
				I_1, 1, 2_	8.20	1.78	10.00	5.00
		I_1, 2_	8.33	I_1, 2, 1_	7.90	1.76	10.00	5.00
				I_1, 2, 2_	9.10	1.22	10.00	6.00
				I_1, 2, 3_	8.70	1.19	10.00	6.00
				I_1, 2, 4_	7.80	1.40	10.00	6.00
		I_1, 3_	7.72	I_1, 3, 1_	7.50	2.01	10.00	3.00
				I_1, 3, 2_	7.90	1.81	10.00	4.00
				I_1, 3, 3_	8.40	1.28	10.00	6.00
		I_1, 4_	8.31	I_1, 4, 1_	8.40	1.36	10.00	6.00
				I_1, 4, 2_	7.80	1.94	10.00	3.00
I_2_	8.01	I_2, 1_	7.90	I_2, 1, 1_	7.90	1.22	10.00	6.00
				I_2, 1, 2_	7.90	1.37	10.00	6.00
		I_2, 2_	8.12	I_2, 2, 1_	8.30	1.19	10.00	6.00
				I_2, 2, 2_	7.50	1.96	10.00	4.00
		I_2, 3_	7.99	I_2, 3, 1_	8.10	1.76	10.00	5.00
				I_2, 3, 2_	7.60	2.15	10.00	4.00

Researchers across disciplines like ethnology, art, and tourism have acknowledged the unique and seminal cultural essence of intangible heritage as exemplified by Chinese drama for over a century. This longstanding recognition has fueled extensive appreciation and exploration of the profound humanistic value embedded within theatrical heritage sites. However, the current assessment indicates tourism development initiatives connected to these drama heritage sites are still lacking and warrant strategic enhancement. Closing this development gap underscores the urgency of confronting existing hurdles hampering the transmission and advancement of theatrical intangible cultural heritage—thus reinforcing earlier dialogues examining remedies to such challenges.

While the humanistic value aspect has seen considerable progress, there is an urgent need to improve the tourism development dimension to ensure the sustainable preservation and growth of this heritage. Among various aspects of tourism development, infrastructure improvement at tourism sites is deemed crucial [52]. Key elements such as transportation, accommodation, catering, and medical facilities are pivotal in the development of scenic spots and in attracting tourists. This viewpoint is endorsed by international scholars, including Seetanah et al. [53] and Pearce and Wu [54]. The future focus for developing drama-related intangible cultural heritage tourism should be in western Sichuan, where the region's underdeveloped infrastructure currently presents significant challenges.

## 5 Conclusion, policy implications, limitations, and recommendations for future studies

### 5.1 Conclusion

This study aimed to establish an expert-driven evaluation system to assess the sustainable tourism development of drama-related intangible cultural heritage in the Sichuan and Chongqing regions of China. Using hierarchical analysis modeling informed by a survey of 52 experts, the relative weights and current development levels across four dimensions were analyzed: humanistic value, project quality, tourism development, and sustainability. The results reveal that humanistic value is regarded as the most important dimension, carrying 41.70 % weight, indicating the paramount need to preserve the cultural essence and diversity of drama heritage. Project quality ranks second in importance, speaking to the necessity of high-quality tourism programming to attract visitors. Tourism development and sustainability follow, still carrying significant weight in enabling the commercial viability and longevity of this heritage form. At the indicator level, aesthetic appeal, dissemination reach, and site climate conditions emerge as vital for tourism promotion. The ideological value embodied in the dramas themselves is seen as the crucial individual factor, underlining the art form's profound cultural significance. An evaluation of current development conditions shows that while humanistic values are being well preserved, deficiencies in tourism infrastructure in particular pose barriers to sustainable growth.

### 5.2 Policy implications

The findings from this study lead to several implications that can inform policymaking aimed at promoting the sustainable tourism development of drama-related intangible cultural heritage. Firstly, as the results indicate a deficiency in tourism infrastructure, there is a need for policies driving infrastructure upgrades, particularly regarding transportation networks, accommodation facilities, dining establishments, and medical services around intangible cultural heritage destinations. Such policies could include government tax incentives, funding partnerships to support community development projects, and community engagement programs to co-design and stimulate infrastructure investments. This is essential to enable tourism growth and support longer visitor stays.

Secondly, promotional campaigns to enhance visibility and the aesthetic appeal of dramatic heritage sites are crucial to increase their profile and attract greater visitor numbers. This can be achieved by leveraging mass media platforms, arranging cultural arts festivals centered around drama performances, and synergistic collaborations with industries like film and media to integrate drama into popular entertainment. Lastly, it is vital that educational programming and cultural policies emphasize preserving the core ideological values and humanistic essence of the art form, authenticating these dimensions that distinguish the cultural heritage. Local cultural departments must tailor their strategies to balance supporting viable tourism models that provide livelihoods while preventing over commercialization or dilution of the fragile intangible heritage.

### 5.3 Study limitations

While the study contributes valuable findings, it possesses some limitations worth acknowledging. Firstly, the small sample size of 52 surveyed experts restricts the ability to make broader generalizations, warranting expanding the pool of experts in future work for greater representative data. Secondly, the research lacks longitudinal data that would have enabled assessing developmental changes over time. Thirdly, the regional focus only on the Sichuan and Chongqing provinces limits the ability to extend the applicability of the assessment scale and indicators to other geographical and cultural contexts. Fourthly, the reliance on subjective perceptions of development levels by surveyed experts may introduce issues with personal biases. Finally, given the complex and nuanced nature of preserving intangible cultural heritage, quantitative metrics may fail to capture some intricate and context-specific qualitative factors relevant for evaluation.

### 5.4 Recommendations for future research

To build on the study, several promising directions for future research can be identified. Firstly, the analysis approach here could be expanded through longitudinal applications with larger, more diverse expert samples over time to empirically validate the indicators that emerged as most salient from this initial investigation. Secondly, conducting comparative studies across regions and heritage types would determine contextual differences in preservation practices and needs, contributing to a standardized assessment framework. Thirdly, qualitative examinations of tourism's impact on local host communities are needed to offset this study's reliance on quantitative metrics alone. Fourthly, there is scope for establishing integrated quantitative indices to directly measure policy outcomes in terms of balancing preservation with tourism development. Finally, investigating how other developing countries with tensions between economic development drives and heritage conservation manage these through legislation and planning would provide wider learnings to guide sustainable management globally.

Overall, while possessing certain limitations, the current work provides an inaugural foundation and springboard to advance both applied and academic understanding of how to effectively leverage tourism for safeguarding vulnerable intangible cultural heritage traditions through evidence-based policy formulation.

## Funding support

This study is financially supported by the Taishan Young Scholar Program (tsqn202103070), the Taishan Scholar Foundation of Shandong Province, China.

## Data availability statement

The data that supports the findings of this study are available from the corresponding authors upon reasonable request.

## Ethics approval and consent to participate

This research was reviewed and approved by the ethical committee for scientific research at the School of Economics, Shandong University of Technology.

## CRediT authorship contribution statement

**Xi Zhao:** Software, Formal analysis, Conceptualization. **Ehsan Elahi:** Writing – review & editing, Visualization, Validation, Supervision, Resources, Investigation, Funding acquisition. **Fushuai Wang:** Formal analysis. **Hu Xing:** Software, Resources, Data curation. **Zainab Khalid:** Writing – review & editing, Formal analysis.

## Declaration of competing interest

The authors declare that they have no known competing financial interests or personal relationships that could have appeared to influence the work reported in this paper.
