# Supplementary material for: Sustainable tourism development for traditional Chinese drama's intangible cultural heritage
Source: Heliyon. 2024 Jan 30;10(3):e25483. doi: 10.1016/j.heliyon.2024.e25483 (PMC10850591; doi:10.1016/j.heliyon.2024.e25483)
Supplement: Multimedia component 1 [file mmc1.pdf]

Form to confirm authorship changes for *Heliyon*

This form must be ***signed by all authors*** when there is a change in authorship which includes changes to any of the following items: author name(s), order of the authors, the corresponding author(s), the addition of authors, the removal of authors and changes in affiliation.

By personally signing this note, **all** authors confirm that: I) the changes are in accordance with their scientific contribution, II) they agree with all the changes and III) confirm that the authorship list conforms to the authorship criteria outlined on *Heliyon*'s ethics page. IV) it is the responsibility of the corresponding author to get the signature from all co-authors accepting the change. In case of any ethic violation/malpractice in the signature, the corresponding author is accountable. The completed form should be returned along with the final/revised manuscript to proceed further with the manuscript. Manuscripts for which incomplete forms have been submitted will be rejected within 5 working days.

Any disputes on the authorship list and contributions need to be resolved by the involved scientists and *Heliyon* will only proceed with the evaluation of the manuscript once we receive confirmation, through this form, that such an agreement between the authors has been reached.

***Heliyon* will not accept changes to the authorship list in the late stages of the editorial process (when a paper is in Accept in Principle stage, acceptance or after publication)**

Manuscript number: HELIYON-

Article title:

Complete new author list:

Date:

#	First name	Last name	Dept. & Institution name	Institutional email address	Order change (Y/N)	Addition / Deletion	Change in Author name (Y/N)	Affiliation Change (Y/N)	Reason for the change	Signature
1	xi	Zhao	School of Communication Sciences and Arts, Chengdu University of Technology	zhaoxi6291 @outlook.com	N	N	N	N	N	[signature]

2	Ehsan	Elahi	School of Economics, Shandong University of Technology	ehsanelahi @cau.edu.cn	N	N	N	N	N	EHSANELAHI
3	Fushuai	Wang	Moscow School of Economics, Lomonosov Moscow State University	mr_leo_wong @outlook.con ity	N	N	N	N	N	[illegible]
4	Hu	Xing	School of Humanitie s, Geely University of China	xinghu1986 @outlook.co	N	N	N	N	N	[illegible]
5	Zainab	Khalid	School of Economics & Managemen Southeast University	zainabkhalid @seu.edu.cn	N	additoin	N	N	review and editing	[illegible]
